# Observations on the Brumal Retreat of the Swallow

**Published:** 1810-09

**Authors:** 


### Observations on the Brumal Retreat of the Swallow.

As Mr. Steele (see p. 187 of this Number) appears to be anxious to establish the truth, and to obviate unfair objections on every point in Natural History, we are much gratified in just now having it in our power to give what we consider as the most *accurate* opinion on the hibernation of Swallows. The ingenious Author on this subject is well known to us; and as he has given his Readers a list of the authors he has consulted, with references to the particular pages, no fewer than sixty-seven authors, we trust that his inferences will decide the question to the satisfaction of the public. We extract only those passages which appear to bear directly on the point at issue.

"There is, perhaps, no subject in Natural History, which has more engaged the attention of Naturalists, in all ages, than the brumal retreat of the Swallow, neither is there any subject on which more various and contrary opinions have been entertained. Some have supposed that they retire at the approach of winter to the inmost recesses of rocks and mountains, and that they there remain in a torpid state until spring. This was certainly the opinion of Pliny, who says, *Abeunt et hirundines hybernis mensibus; sola carne vescens avis ex iis quæ aduncos ungues non habent; sed in vicina abeunt, apricos* secutæ montium recessus,*

---

"* Some editions have Africos, not apricos. The latter, however, is certainly the best, for Pliny would surely have applied the adjective to montium, and not to recessus. Thus he would have said, Africorum secutæ montium recessus; or else, Africæ montium secutæ recessus.

*cessus, inventæque jam sunt ibi nudæ atque deplumes.* Lib. x. cap. xxiv.

"But notwithstanding that we have the authority of so learned, though at the same time so credulous, a Naturalist as Pliny, it seems almost absurd to suppose that the swallow differs so much in its nature from other birds, as we do not find any material difference either in its external or internal formation. Others have conjectured that these birds immerse themselves in the water at the approach of winter, and that they remain at the bottom in a state of torpidity, until they are again called forth by the influence of the vernal sun. Linnæus was probably of this opinion, when he said, *Hirundo Rustica habitat in Europæ domibus intra tectum, unaque cum urbica Autumno demergitur, vereque emergit.* But most likely in this, as in some few other cases, he gave credit to the fabulous assertions of others, without examining into the truth of them himself.* There are several instances on record of their having been found in such situations clustered together in great numbers, and that, on being brought before the fire, they have revived and flown away. But unfortunately, few of these accounts have been well authenticated, and the celebrated Mr. John Hunter has clearly proved, from various experiments which he himself made, that these birds cannot continue long under water without being drowned. I do not mean to deny that swallows may have *occasionally* been found under water; for it is well known that they have, and this probably has given rise to the absurd notion entertained by some, that the whole of the species winter in that element. But I should certainly attribute their being found in such situations to mere accident; and I think it might have been occasioned by some such circumstance as the following:

"It is well known that, towards the latter end of autumn, swallows frequently roost by the sides of lakes and rivers;† we will suppose, therefore, that a number of these birds had retired to roost on the banks of some shallow and muddy river at low tide, and that they had been induced by the cold to creep among the reeds or rushes

---

* It has been doubted by some, whether Linnæus meant any more, by demergitur and emergit, than that the Swallow was hid, and came forth again.

" † This circumstance may have contributed to induce some persons to believe that they go into the water.

which might grow in the shallow parts of the river, and that while in this situation, driven into a state of torpidity by the cold, they had been overwhelmed, and perhaps washed into the current, by the coming in of the tide.

"Having thus accounted for the manner in which swallows might chance to get into the water, it remains to be considered by what means they may have been sometimes taken out *alive*. Let us suppose therefore that some fishermen, as is very likely to be the case, had availed themselves of the coming in of the tide to catch fish, and that the swallows, which we have before supposed to have been carried into the current, coming in contact with their nets, were consequently drawn out of the water by them, and, not having been long under water, were not completely drowned.

"There are several other circumstances which seem to favour the opinion, that these birds remained concealed during winter in this Country. Among others, the most striking is, that swallows, *Hirundines Rusticæ*, as well as martins, *Hirundines Urbicæ*, have sometimes appeared very late in Autumn, a considerable time after they were all supposed to have taken their departure.* Again, as I have before had occasion to observe, they have sometimes been taken out of the water, in winter, in a torpid state, not only out of rivers, but also out of lakes and stagnant pools, and even out of bogs.† They have likewise been found concealed in the crevices of rocks, in holes of old decayed trees, in old ruined towers, and under the thatch of houses.‡

"From the consideration of the above facts alone, without

* Of this we have several instances: Bewick, in his History of British Birds, Introduction, p. xvii. takes notice of having seen a straggling swallow so late as the end of October; and Mr. White, in his Natural History of Selborne, mentions having seen a house martin flying about in the month of November. To which I may add, that in the year 1804, I saw several, both martins and swallows, flying about in the neighbourhood of London, as late as October the 19th.

† For further particulars relative to the torpidity of swallows, see Miscellanies, by the Hon. Daines Barrington, page 225 and Sequel; also Buffon, Hist. Nat. des Oiseaux, 4to. Paris, 1780, Plan d'Ouvrage, p. xiii.

‡ A great many sand martin's holes have been opened in Winter, and nothing has been found in them but old nests.—See Phil. Trans. vol. 51, p. 463.

without making any further enquiry into the subject, many persons have concluded that the whole tribe always winter in similar situations. It seems however much more probable, that those birds, which may have been found in a state of torpidity, as above described, had, owing to some accident, been hatched later in the year than ordinary, and that consequently they had not acquired sufficient strength to undergo the fatigue of a long journey upon the wing, at the time when the migration of the rest of their species took place. It is very probable, that many of these, in order to shelter themselves from the inclemency of the weather, may have retreated to holes of rocks, &c. where from cold and hunger they may have sunk into a state of torpidity.* Others, for the same reason, may have crept among the weeds, which grow by the sides of rivers and ponds, where they may have been overwhelmed by the increase of the water, occasioned by the heavy rains which often happen towards the end of Autumn, and some, which may not have been long immersed, may probably have been restored to life, when brought into the sun or before a fire.

" But that the chief part of each species migrate is so well established by a multitude of corresponding facts, that it seems almost an absurdity to doubt of it. In the first place I would observe, that if these birds lay concealed in winter, in the same countries which they inhabit in summer, they would probably make their first appearance in spring, in mild weather, and would most likely appear sooner in early than in late seasons, which is quite contrary to experience. For several years past I have observed that chimney swallows have appeared first in cold weather. I have sometimes seen them as early as April the 2d, when the mercury in the thermometer has been below the freezing point. On the other hand I have often taken notice, that during a continuance of mild weather for the space of a fortnight, in the month of April, not so much as one swallow has appeared.

" It is a well known fact that the swallow, like most other birds of passage, appears earlier and departs later in

* See Montagu's Ornithological Dictionary, under the word *Martin*.

It is by no means improbable, that very cold and frosty weather in Spring, may sometimes drive the swallow, just arrived, into some snug retreat, where it may remain until the warm weather returns.—See Phil. Trans. vol. 65, p. 259.

in the Southern than in the Northern parts of Great Britain; and it must have been observed, by every one who is attentive to Natural History, that towards the latter end of September, swallows, *Hirundines Rusticæ*, as well as martins, *Hirundines Urbicæ*, congregate in great numbers, and are frequently seen sitting on the tops of houses, and on rocks near the sea. These meetings usually continue for several days, after which they suddenly disappear.

" Swifts, *Hirundines Apodes*, also begin to assemble previous to their departure: early in July, their numbers daily increase, and large bodies of them appear together: they soar higher in the air, with shriller cries, and fly differently from their usual mode. These meetings continue till towards the middle of August, after which they are no more seen.*

" Sand Martins, *Hirundines Ripariæ*, likewise flock together in Autumn. Some years ago they appeared in such numbers in London and its neighbourhood, that hundreds were, every day, caught by boys in the streets, for nearly the space of a week.

" From all the above mentioned circumstances, as well as from the great length of the wings, in proportion to the bulk of the body, of all this genus,† it must appear evident that swallows are birds of passage: for it is hardly to be supposed that they would assemble together merely to hide themselves; on the contrary, it is most probable that, were this the case, each individual bird would seek a hiding place for itself.

" It will be proper now to examine the accounts of mariners and others, who have seen these birds on their passage, many hundred miles out at sea, and on whose ships they have alighted to rest, almost exhausted with fatigue and hunger, by which means we may be enabled, in some measure, to determine to what quarter of the globe they retire, when they leave Europe in Autumn.

( To be continued. )

---

* See Bewick's British Birds, p. 261.

† If we calculate the velocity of this bird on the wing, and that it can and does suspend itself in the air for fourteen or fifteen hours together in search of food, it cannot fly over a space of less than two or three hundred miles in that time. Montagu Ornith. Dict.

